# Who is crying wolf? Seasonal effect on antipredator response to age-specific alarm calls in common ravens, *Corvus corax*

**DOI:** 10.3758/s13420-020-00455-0

**Published:** 2021-01-08

**Authors:** Mario Gallego-Abenza, Christian R. Blum, Thomas Bugnyar

**Affiliations:** 1 Department of Behavioural and Cognitive Biology, University of Vienna, Vienna, Austria; 2 Konrad Lorenz Forschungsstelle, Core Facility for Behaviour and Cognition, University of Vienna, Grünau im Almtal, Austria

**Keywords:** Discrimination, Perception, Communication, Antipredator behaviour

## Abstract

Communication about threats including those posed by the presence of predators occurs mainly through acoustic signals called alarm calls. The comprehension of these calls by receivers and their rapid antipredator response are crucial in terms of survival. However, to avoid overreaction, individuals should evaluate whether or not an antipredator response is needed by paying attention to who is calling. For instance, we could expect adults to be more experienced with predator encounters than juveniles and thus elicit stronger antipredator responses in others when alarming. Similarly, we could expect a stronger response to alarm calls when more than one individual is calling. To test these assumptions, we applied a playback experiment to wild ravens, in which we manipulated the age class (adult or juvenile) and the number (one or two) of the callers. Our results revealed a seasonal effect of age class but no effect of number of callers. Specifically, the ravens responded with stronger antipredator behaviour (vigilance posture) towards alarm calls from adults as compared to juveniles in summer and autumn, but not in spring. We discuss alternative interpretations for this unexpected seasonal pattern and argue for more studies on call-based communication in birds to understand what type of information is relevant under which conditions.

**Supplementary Information:**

The online version contains supplementary material available at 10.3758/s13420-020-00455-0.

## Introduction

Birds are famous for their vocalization. Song learning, for instance, has been intensively studied over the last decades (Catchpole & Slater, 2008) and is fairly well understood from a behavioural and neurobiological perspective (Bolhuis & Gahr, 2006), making it an excellent model for human speech (Bolhuis, Okanoya, & Scharff, 2010). In comparison to the vast literature on song learning, bird calls have received limited attention (Marler, 2004), and studies on the cognitive skills underlying the production and usage of bird calls are rare. In respect to the latter, research on a single grey parrot, ‘Alex’, has become famous: using English words for communicating with human trainers, Alex not only labelled objects, but responded to questions probing his knowledge (e.g. of relational concepts like same/different) and expressed intentions via requests (Pepperberg, 1999). While Alex’s skills are impressive in many ways, sparking debates on various conceptual and methodological levels (Pepperberg, 1983, 1990, 2008), his apparent understanding and intentional use of communicative signals with humans raises the question of what predispositions these skills might be based upon? Why would a grey parrot like Alex need a sophisticated neuro-cognitive machinery allowing him to copy sounds, attach meaning to it, form concepts, and use them in interaction with others? Twenty-five years of research on grey parrots´ life support the idea of evolutionary pressures underpinning complex communicative and cognitive capacities (Pepperberg, 2002). It has been argued that parrots need such abilities in daily social life (Pepperberg, 1999), which in the case of Alex, happened to be the human setting. But what challenges could parrots, or other birds, face under ecologically relevant situations that require communication other than song, i.e. that they should ‘talk’ about?

Obvious candidates are live-threatening events, like predator encounters, that can occur to wild animals at any time. Using communication may help individuals to detect predators (Smith, 1965; Zuberbühler, Noë, & Seyfarth, 1997) and assess the type or level of threat (Seyfarth, Cheney, & Marler, 1980), and, as a consequence, respond with appropriate behaviours such as escaping, hiding or repealing an attack (Botham et al., 2008; Kotler, Blaustein, & Brown, 1992; Lohrey, Clark, Gordon, & Uetz, 2009). Potential victims may also gather forces and drive the predator away from the area (Foster & Treherne, 1981). While acoustic signals given in the presence of predators are commonly referred to as alarm calls (Hauser, 1996), the behaviour associated with driving the predator away is known as mobbing or collective anti-predator behaviour (Curio, 1978; Graw & Manser, 2007).

Like many mammals, birds tend to give different alarm calls to specific events in the environment, like the occurrence of ground or areal predators (Evans, Evans, & Marler, 1993). Avian alarm calls are thus a prime candidate for investigating information content about external reference (Gill & Bierema, 2013). Experiments revealed that in some species, alarm calls denote different types of predator classes that require different response strategies (Kalb, Anger, & Randler, 2019; Suzuki, 2012, 2014), which fulfil the criteria of functional reference (Evans et al., 1993; Rendall, Owren, & Ryan, 2009); in other species, however, the calls denote the urgency level to respond (Leavesley & Magrath, 2005), and thus likely represent differences in arousal states (Blumstein & Récapet, 2009). Typically, alarm calls have a strong genetic component in respect to production, but are relatively flexible in respect to usage (Fichtel & Van Schaik, 2006; Townsend, Rasmussen, Clutton-Brock, & Manser, 2012). Senders may thus fine-tune the use of alarm calls, for example to denote a specific predator type or behaviour (Griesser, 2008; Suzuki, Wheatcroft, & Griesser, 2016), and/or adjust their signalling to the audience, for example call more when kin or mating partners are present (e.g. Shields, 1984). On the receiver side, getting accurate information about predators and learning to respond appropriately to alarm calls are of high survival value (Griesser, 2013). Receivers may readily learn about alarm calls even across species, as demonstrated in the mobbing flocks of mixed-species communities of songbirds (Magrath, Pitcher, & Gardner, 2009; Wheatcroft, Gallego-Abenza, & Qvarnström, 2016).

Like most vocalizations, alarm calls also contain information about the sender, such as its sex, age class, kin or individual identity (Blumstein & Munos, 2005). Receivers of alarm calls may thus not only respond to the type of threat/level of urgency encoded in the calls but take the senders’ features and/or identities into account when engaging in antipredator behaviour (Hare, 1998; Hare & Atkins, 2001). Surprisingly few studies have tested the receivers’ responses to such sender-specific characteristics in birds (with the exception of kin in nepotistic alarm calling and/or mobbing, e.g. Griesser & Ekman, 2004, 2005). Experiments on Pied flycatchers (*Ficedula hypoleuca*) showed that they do not automatically respond to any alarm calls of their territory neighbours with predator mobbing but selectively help those neighbours to mob a predator, who had helped them before (Krams, Krama, Igaune, & Mänd, 2008; Wheatcroft & Price, 2008). The reciprocal pattern indicates that the birds acquire some form of knowledge and/or attribute about their neighbours through previous predator encounters. Recent experiments on jackdaws (*Corvus monedula*) revealed that birds respond stronger with collective anti-predator behaviour to the play back alarm calls of colony members as compared to non-colony members, indicating that receivers discriminate between familiar and unfamiliar birds (Woods, Kings, McIvor, & Thornton, 2018). Furthermore, the number of callers had a similar positive effect on the probability to engage in collective antipredator behaviour, indicating that receivers take into account whether the alarm calls were elicited by a single or a few birds and hence the intensity of the response (Coomes, Mcivor, Thornton, Coomes, & Thornton, 2019). Such assessment capability by receivers was also documented in small mammals, precisely in adult Richardson´s ground squirrels (Sloan & Hare, 2008). When confronted with alarm calls from conspecifics and closely related heterospecifics during foraging, carrion crows tended to respond to any alarm calls (Bílá, Beránková, Veselý, Bugnyar, & Schwab, 2017), whereas ravens adjusted their antipredator behaviour depending on the perceived risk (whether or not they snatched food from predators; Nácarová, Veselý, & Bugnyar, 2018) and the familiarity of the calling species (Davidkova, Veselý, Syrova, Nacarovà, & Bugnyar, 2020).

In the present study, we followed the logic of the studies on jackdaws (Coomes et al., 2019) and investigated whether common ravens are attentive to sender-specific characteristics in alarm calls. Unlike jackdaws, adult ravens defend large territories (Scarpignato & George, 2011) and thus do not form colonies during breeding. However, non-breeding ravens tend to form large groups, usually near food sources (Heinrich, 1989; Loretto, Schuster, & Bugnyar, 2016). These groups are composed mainly of immature individuals (juveniles in their first year, subadults in their second or third year; making up about 20% and 60% of a group, respectively) but also of adult birds (older than 3 years, often having no partner and/or no territory; typically about 20% of a group) (Braun & Bugnyar, 2012; Heinrich, 1989). Apart from age-class, raven foraging groups are structured by social relationships (social bonds based on reciprocal exchange of affiliative interactions; Braun & Bugnyar 2012) and kinship (Szipl, Ringler, & Bugnyar, 2018; but see Parker, Waite, Heinrich, & Marzluff, 1994). Yet, the foraging groups have an open character, with individuals coming and going on a regular basis (Heinrich, 1989). How long birds stay at a site/in a given group varies extensively from a few days to years; hence, individuals can meet rarely, regularly or frequently at the same or different sites (Loretto et al., 2017). Taken together, the socially structured but fluid nature of raven foraging groups constitutes a promising scenario for studying what individuals know about others and which features they attend to (Boucherie, Loretto, Massen, & Bugnyar, 2019). With respect to alarm calls, we could expect adults to be more reliable in terms of threat perception than young individuals, due to the adults’ previous life-experience encountering different types of threats. Furthermore, we could expect not only the number of callers but also the identity of the callers to be critical for the receivers’ decision to engage in antipredator behaviour.

Here we focussed on two of the identified factors, age class (as a proxy for experience) and number and callers (as a proxy for threat intensity), while controlling for the callers’ identity (always unfamiliar). We exposed groups of free-ranging ravens during foraging to playbacks of a standardized number of alarm calls given either by a single juvenile, two juveniles, a single adult or two adults. We predicted ravens to show stronger responses when listening to adult individuals as compared to juveniles and when listening to two different individuals alarming as compared to one individual.

## Material and methods

### Study site and study species

This study was conducted at the Cumberland Wildpark, a zoo in the Austrian Alps (N 47°48.421′, E 13°57.032′), where groups of common ravens snatch food from animal enclosures. These ravens are the focus of a long-term monitoring program (started in 2007), during which more than 300 birds have been marked with rings and wing tags for individual identification. The size of the daily foraging groups in the park ranges between 20 and 80 individuals. The groups are composed mostly of non-breeders in the first years of life (juveniles and subadults, < 4 years old) but also adults that do not hold a territory and/or visit this group in the non-breeding period opportunistically; they continuously change in composition with noticeable individual differences in terms of how long ravens stay and/or leave (Braun & Bugnyar, 2012; Loretto et al., 2017). We focused on the wild boar enclosure for our experiment, as this enclosure allows a good view of the foraging ravens, the wild boars themselves do not represent a risk for the ravens, and the ravens are known for responding well to playbacks of heterospecific and conspecific calls at this location (Nácarová et al., 2018). A total of 48 trials were conducted in three different seasons, starting in spring 2019 (17 January 2019–7 May 2019), followed by autumn (25 September 2019–18 December 2019) and finishing in summer 2020 (25 May 2020–18 July 2020), with two non-testing periods of approximately 4 and 7 months between seasons.

### Playback stimuli

We used alarm calls that were recorded from captive ravens at Haidlhof Research Station, which is located in the east of Austria, about 200 km away from our field site in the Alps. While our marked wild ravens may roam over larger areas (Loretto et al., 2017), they have never been observed near Haidlhof.

**Fig. 1:**
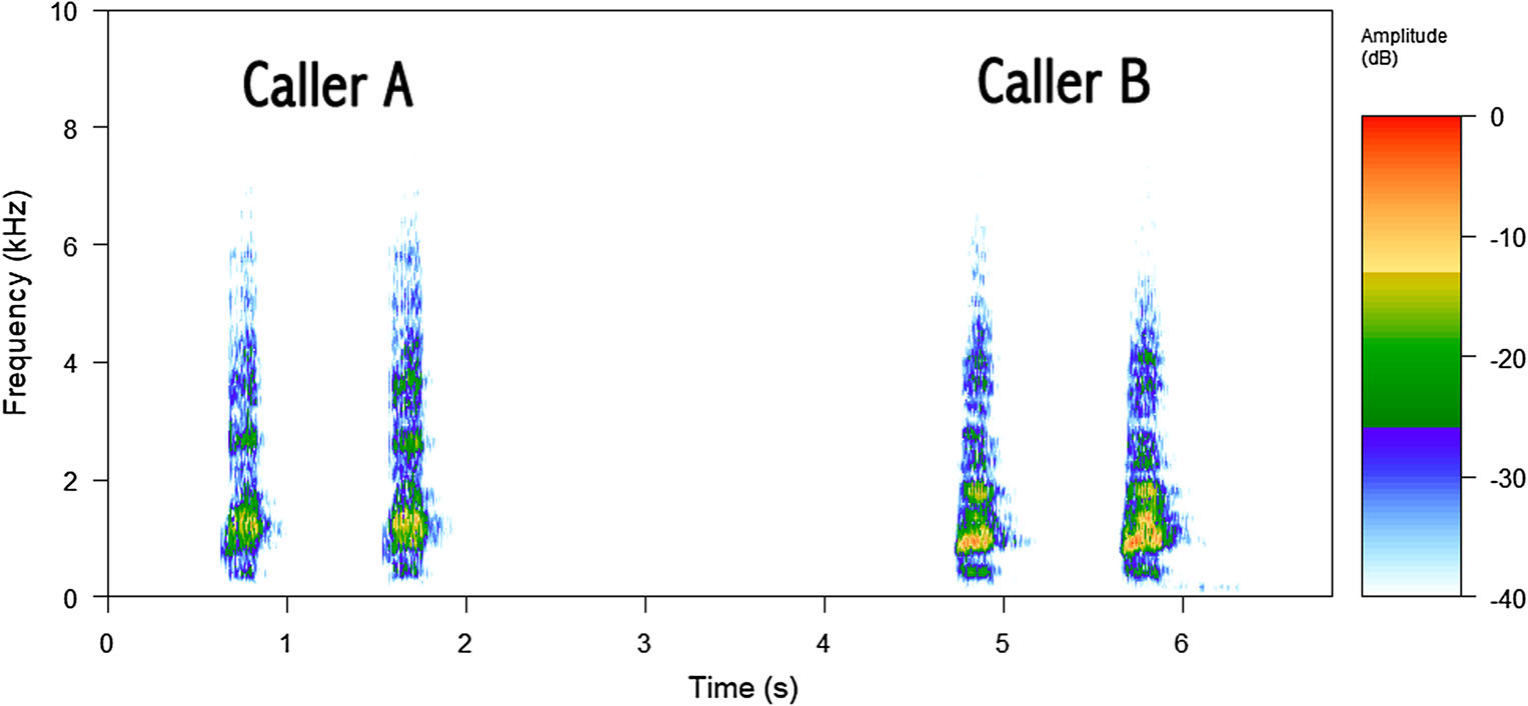
Spectrogram of a single playback stimulus. In this case, two different adult individuals were broadcast

Ravens at Haidlhof were housed in a social group structured by age class (juveniles, subadults and adults) simulating the wild conditions. In an experimental study, these ravens were exposed to a human carrying a dead raven resulting in intense mobbing behaviour and alarm calling (Blum, Fitch, & Bugnyar, 2020). We used these calls from that experiment because: (1) these captive ravens were unfamiliar to the wild ravens in Grünau, and (2) we could identify callers at the individual level. The known identity of callers allowed us to compose the four different treatments: single caller versus two callers from either juvenile or adult age class, thus creating 16 different broadcasting files (four per each treatment) to be broadcast in randomized order within and among each season, conducting 16 trials per season, 48 trials in total. Testing days were separated from each other by 4.3 days on average (range 2–18) to avoid habituation. Sex was also known and controlled within a treatment composition, generating a similar number of broadcasting files of each sex. Each treatment was composed using four calls, where the third and fourth calls occurred after 3 s of silence interval and could correspond to either the same or a different individual (see Fig. 1). We equalized all calls´ amplitude in the composed files to be broadcast using Audacity software (https://www.audacityteam.org/). Alarm calls were played back in .wav format using a digital music player (Musrun k188) connected to a loudspeaker (JBL xtreme, frequency response 70–20,000 Hz). All calls were standardized to an identical volume of 73 dB measured at 2 m of distance (Sound Level Meter RadioShack, model 3300099, A-weighting, fast response).

### Behavioural responses

Playbacks were conducted during the feedings of wild boars, i.e. while the wild ravens were foraging. The same experimenter (MGA) conducted all trials to avoid a potential effect of experimenter identity (MGA has been studying the ravens at this location for than 2 years: hence, the ravens were well habituated to his presence from the beginning of the experiment). Ravens´ responses were filmed using two GoPro Hero 5 cameras from fixed positions at 2 m and 5 m of distance to the foraging site. We measured the total number of ravens present in the camera´s field of view. In addition, we scored whether ravens in the video were flying off from the foraging place or adopted a vigilance posture for 5 s right after broadcasting the alarm calls. We defined vigilance posture as being when ravens raised up their heads, elongating their necks, and directed their gaze repeatedly towards sky, while switching between eyes, following studies on antipredator behaviours conducted in other avian species (Fernández-Juricic, 2012; Guillemain, Duncan, & Fritz, 2001).

### Statistical analyses

We conducted our statistical analyses in R software (v. 3.6.1., R Development Core Team 2014). For modelling the two response variables: `vigilance posture´ and `flying off´, we used the function glmer in the package `lme4´ (Bates, Mächler, Bolker, & Walker, 2015). Due to an inconstant number of total ravens across testing days, we used the command `cbind´ within the model formula to control for it, thus modelling the proportion of ravens showing any of the two behavioural responses (`vigilance posture´ and `flying off´) with a binomial error distribution. To answer the question whether ravens responded differently depending on the `treatment´ (single adult, two adults, single juvenile or two juveniles) being exposed to, we used `season´ as random factor, together with `broadcasting file´, controlling for potential seasonal effects. When testing the effect of `season´ on ravens´ response, we included it as fixed factor and ´broadcasting file´ remained as unique random factor. When using `age class´ or `calling composition´ (one or two callers), trials were clumped together according to these predictors. Model selection through both AICc and weight comparison was conducted using the function `model.sel´, ´MuMIn´ package (Barton, 2019). The best model explaining `vigilance posture´ response contained the interaction between `age class´ and `season´, in order to examine significant differences within each season, we conducted a post hoc Tukey contrast test using `emmeans´ package (Searle, Speed, & Milliken, 1980) to calculate differences in estimated marginal means and P values.

## Results

**Fig. 2:**
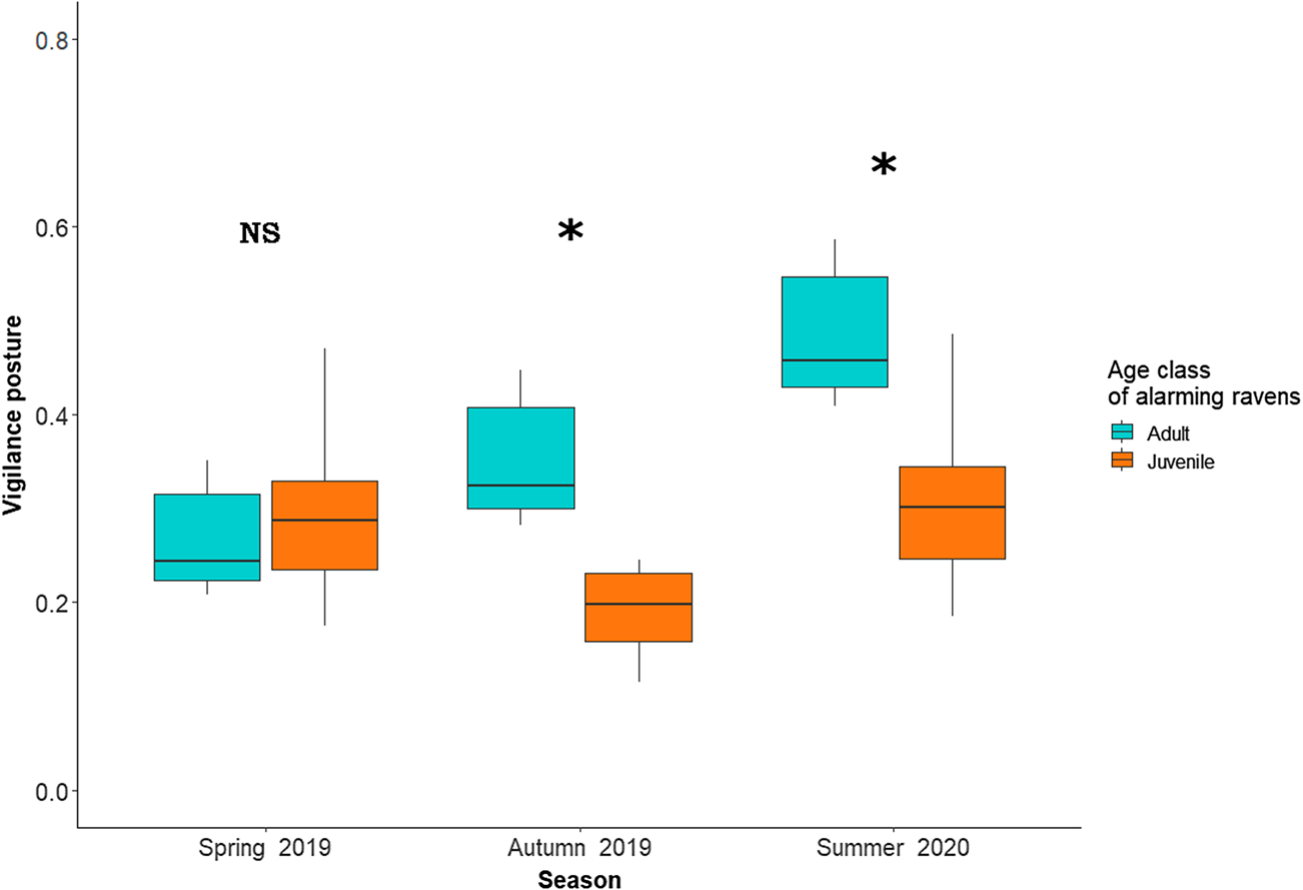
This plot shows the significant differences (post hoc Tukey contrast test, using “emmeans” package) found in the proportion of ravens responding towards adult and juvenile alarming conspecifics in the three tested seasons

Ravens responded to played back alarm calls by adopting `vigilance posture´, in 46 out of 48 trials (95.8%), whereas a `flying off´ response occurred in only 12 out of 48 trials (25%). For both behavioural response variables, the model containing `treatment´ as unique explanatory predictor did not result in a significant difference between the four playback conditions (single adult, two adults, single juvenile, two juveniles). However, model selection indicated that for `vigilance posture´ the model containing the interaction between `season´ and `age class´ was the best model (lower AICc and higher weight; see Tables 1 and 2). `Age class´ independently affected ravens´ vigilance response, where ravens were less responsive to juvenile compared to adult callers (Estimate = -0.777, SE = 0.342, Z = -2.272, P = 0.023). Similarly, we found that `season´ had an effect on the ravens´ vigilance posture response to any played-back alarm call´s composition, whereby higher vigilance posture values occurred in summer (Estimate = 0.563, SE = 0.259, Z = 2.172, P = 0.029). Additionally, the interaction between the two abovementioned factors revealed age-specific responses depending on the season. The post hoc Tukey contrast test revealed that stronger responses to adult as compared to juvenile callers occurred in summer and autumn, but not in spring (Fig. 2).

**Table 1:** Model selection with model candidates to explain the vigilance posture response ordered by AICc and weight

Explanatory variables	df	logLik	∆AICc	Weight
Age class x Season	7	-128.26	0	0.651
Season	4	-133.01	1.81	0.263
Age class	3	-136.15	5.52	0.041
Null model	2	-138.03	7.00	0.020
Season x number of callers (1 or 2)	7	-132.15	7.78	0.013
Number of callers (1 or 2)	3	-137.91	9.05	0.007
Treatment	5	-135.96	10.03	0.004
Season x Treatment	13	-126.93	17.24	0.000

**Table 2:** Summary of the generalized mixed model containing the interaction effect of `season´ and `age class´ in the vigilance posture response to broadcasted alarm calls of conspecifics in different seasons

Parameter	Estimate	SE	Z value	P
Intercept	-0.633484	0.231475	-2.737	0.00621 **
Season *Summer*	0.562793	0.259168	2.172	0.02989 *
Season *Spring*	-0.401102	0.289806	-1.384	0.16635
Age class *Juvenile*	-0.776795	0.341911	-2.272	0.02309 *
Season *Summer* x Age class *Juvenile*	-0.002671	0.387085	-0.007	0.99450
Season *Spring* x Age class *Juvenile*	0.895711	0.401529	2.231	0.02570 *

A similar procedure with model selection was followed to estimate how `season´ and `age class´ affected the `flying off´ response. In this case, the model containing the interaction between `season´ and `treatment´ was classified as the best model (Supplementary Table 1). However, neither of those had a significant effect (separately or in interaction) on ´flying off´ response (Supplementary Table 2).

## Discussion

We tested whether wild ravens respond to two types of information possibly encoded in conspecifics alarm calls, i.e. the age class of the caller, and whether calls are given by one or more individuals. Playbacks of alarm calls elicited a stronger vigilance response when given by adults as compared to juveniles in two out of three seasons (in summer and autumn, not in spring). The number of calling individuals, however, did not lead to a significant difference in the ravens´ antipredator responses.

That ravens respond more strongly to alarm calls of adults rather than juveniles meets our expectation and supports the assumption that receivers can extract information about the age class of alarm callers. That this effect is dependent on season was not expected, however, and may be explained in different ways. On the one hand, ravens might have responded less to alarm calls given by juveniles in summer and autumn because at that time juveniles are very young and likely lack experience with predators and/or may easily give alarm calls to any disturbing situation. Hence, juveniles might be perceived as less reliable in alarm calling than adults. Similar findings have been described in some studies on mammals (Ramakrishnan & Coss, 2000; Seyfarth & Cheney, 1986), whereas other studies reported no effect of age class (Swan & Hare, 2008) or even the opposite (Blumstein & Daniel, 2004). On the other hand, ravens might be particularly receptive to alarm calls in spring, when local low temperatures allow them to scavenge on carcasses, putting them into increased contact with predators, and their survival rates are lowest (Webb, Boarman, & Rotenberry, 2004). Hence, in the season with a high likelihood of dangerous predator encounters, they might respond to any alarm calls, irrespective of the caller´s age class. Similar patterns of seasonal variation in antipredator behaviour have been described for other species (Shedd, 1982; Uchida, Suzuki, Shimamoto, Yanagawa, & Koizumi, 2016). Interestingly, the temporal pattern of our results renders either interpretation unlikely (Fig. 2). Receivers did not increase their response to juvenile alarm calls across the year, as would be expected with increasing experience of young birds or with increased threat levels after the first winter; conversely, they decreased their response to alarm calls of adults across the seasons, showing the lowest response rates in spring. This pattern suggests that ravens treat alarm calls of adults particularly seriously during summer and autumn, i.e. the period when families with young ravens are around. Alternatively, the pattern could be interpreted as resulting from reduced attention towards alarm calls of adults during cold periods (winter-spring). Although ravens face severe foraging competition in winter (Heinrich, 1989) and may divide their attention between gaining access to food (B. Heinrich & Marzluff, 1995), fending off conspecific kleptoparasitism and cache pilferage (Bugnyar & Kotrschal, 2002; Gallego-Abenza, Loretto, & Bugnyar, 2020; Heinrich & Pepper, 1998), there are hardly any indications that competition for food affects their antipredator behaviour, at least not at our study site (Nácarová et al., 2018). Finally, the temporal pattern found might be considered an artefact of our testing. Note that the order of playback presentation (first in spring 2019, then in autumn 2019, and then in summer 2020) makes it unlikely that our results are due to an order effect or habituation. Moreover, the played-back individuals were unfamiliar to the tested ravens, indicating that receivers can extract age-class information from any conspecific alarm calls, which is perfectly in line with the ecological relevance of alarm calls (Gill & Bierema, 2013) and the structure of raven foraging groups with moderate to high fission-fusion dynamics (Braun, Walsdorff, Fraser, & Bugnyar, 2012).

Contrary to our expectation and to recent findings in jackdaws (Coomes et al., 2019), we could not find any effect of the number of played-back individuals on ravens’ antipredator behaviour. Our failure to detect a numerical discrimination through alarm calls may be due to the salience of the chosen stimuli. For instance, while we used one or two callers, the study on jackdaws used one, three or five callers; it is known that animals, including birds, have more difficulties in discriminating one versus two in comparison to one versus larger numbers (Tornick, Callahan, & Gibson, 2015). In a study conducted on mammals, more precisely on Richardson´s Ground Squirrels, only adult receivers showed enhanced antipredator responsiveness to two versus one alarm caller, even though juvenile receivers discriminate among individual callers, suggesting a developmental shift in the parameters employed to assess the veracity of any threat (Sloan & Hare, 2008). Alternatively, the ravens might have a problem in picking up on the individual information in the calls. We already know that some ravens’ calls like food-associated calls (`haa´) and territory calls (`rab´) contain strong individual signatures, which the birds respond to in habituation-dishabituation experiments (Boeckle, Szipl, & Bugnyar, 2012); in other calls, like those given in agonistic interactions, individual information is less evident in comparison to affective information (Szipl, Ringler, Spreafico, & Bugnyar, 2017). Possibly, this is similar with alarm calls. A proper acoustical analysis and further playback experiments are needed to investigate this question.

Taken together, our study contributes to our understanding of what type of information birds may pick up when hearing alarm calls. While most studies on alarm calls have focused on functional reference about predators (Evans et al., 1993; Griesser, 2008; Suzuki, 2011, 2014), relatively few studies have experimentally tested for other types of information, like familiarity of caller/group membership (Griesser & Ekman, 2004, 2005; Woods et al., 2018), number of callers and callers´ age class (Coomes et al., 2019; this study). The findings reveal that birds respond selectively to different features that appear to be ecologically relevant, like the seasonal effect of responding to adults in this study. What is yet unknown is how much birds make use of individual information encoded in alarm calls, as several results could be explained by (refined) class-level discrimination (Tibbetts & Dale, 2007). In this respect, studies on behavioural deception are interesting, as there are multiple reports of individual callers becoming unreliable when repeatedly giving false alarms (Flower, Gribble, & Ridley, 2014; Munn, 1986). Experimental approaches manipulating the reliability of alarm callers could be a promising future step.

Coming back to our original question about what birds `talk about´, the information content in alarm calls certainly encompasses only one of many aspects in avian communication. Yet, these studies support the notion that examining the socio-cognitive underpinnings of call-based communication in birds is a promising endeavour (Lambert, Jacobs, Osvath, & Von Bayern, 2019). If we eventually manage to examine the information content (such as individual attributes, motivations, affective states, functional reference to external events) of various calls individuals of a species respond to, we may end up with a relatively complex picture on the receiver side, just as Pepperberg´s pioneering Alex studies defined the realm of be possibility on the production side.

## Supplementary Information

ESM 1 (DOCX 11 kb)

ESM 2 (DOCX 12 kb)

## Data Availability

The data and materials for the experiments will be available upon publication.
