# Supplementary material for: Who is crying wolf? Seasonal effect on antipredator response to age-specific alarm calls in common ravens, Corvus corax
Source: Learn Behav. 2021 Jan 8;49(1):159–67. doi: 10.3758/s13420-020-00455-0 (PMC7979661; doi:10.3758/s13420-020-00455-0)
Supplement: Supplementary file 1 — (DOCX 11 kb) [file 13420_2020_455_MOESM1_ESM.docx]

| Explanatory variables | df | logLik | ∆AICc | Weight |
| --- | --- | --- | --- | --- |
| Season x Treatment | 13 | -48.845 | 0.0 | 0.948 |
| Season x Calling composition | 7 | -61.701 | 5.81 | 0.052 |
| Season | 4 | -86.207 | 46.95 | 0.0 |
| Season x Age class | 7 | -83.854 | 50.11 | 0.0 |
| Null | 2 | -91.783 | 53.44 | 0.0 |
| Calling composition | 3 | -91.062 | 54.27 | 0.0 |
| Age class | 3 | -91.340 | 54.83 | 0.0 |
| Treatment | 5 | -89.714 | 56.46 | 0.0 |

**Supplementary Table 1.** Table containing model selection with models candidates to explain `flying off´ response ordered by AICc and weight.
