# Supplementary material for: Who is crying wolf? Seasonal effect on antipredator response to age-specific alarm calls in common ravens, Corvus corax
Source: Learn Behav. 2021 Jan 8;49(1):159–67. doi: 10.3758/s13420-020-00455-0 (PMC7979661; doi:10.3758/s13420-020-00455-0)
Supplement: Supplementary file 2 — (DOCX 12 kb) [file 13420_2020_455_MOESM2_ESM.docx]

**Supplementary Table 2**. Summary of the generalized mixed model containing the interaction effect of `season´ and `treatment´ in the flying off response to the 4 combinations of broadcasted alarm calls in different seasons.

| Parameter | Estimate | SE | Z value | P |
| --- | --- | --- | --- | --- |
| Intercept | -2.9191 | 0.9277 | -3.146 | 0.00165 |
| Treatment *Two juveniles* | -24.8346 | 51448.1824 | 0 | 0.99961 |
| Treatment *Unique adult* | -2.4721 | 1.8119 | -1.364 | 0.17245 |
| Treatment *Unique juvenile* | 0.5126 | 1.2950 | 0.396 | 0.69224 |
| Season *Summer* | -23.8309 | 72299.9908 | 0 | 1 |
| Season *Spring* | -0.3038 | 0.7523 | -0.404 | 1 |
| Treatment *Two juveniles* x Season *Summer* | 47.4291 | 88736.7124 | 0 | 1 |
| Treatment *Unique adult* x Season *Summer* | 3.1884 | 88276.0053 | 0 | 1 |
| Treatment *Unique juvenile* x Season *Summer* | -1.5327 | 95085.4348 | 0 | 1 |
| Treatment *Two juveniles* x Season *Spring* | 25.8641 | 51448.1824 | 0.001 | 1 |
| Treatment *Unique adult* x Season *Spring* | -19.8680 | 44525.8694 | 0 | 1 |
| Treatment *Unique juvenile* x Season *Spring* | -24.3650 | 46136.9100 | -0.001 | 1 |
